# Supplementary material for: Validation of a risk-prediction model for pediatric post-discharge mortality after hospital admission for infection in Rwanda: A prospective cohort study
Source: PLOS Glob Public Health. 2025 Jul 1;5(7):e0004606. doi: 10.1371/journal.pgph.0004606 (PMC12212559; doi:10.1371/journal.pgph.0004606)
Supplement: S2 Table — (DOCX) [file pgph.0004606.s002.docx]

|  | **Rwanda (N=1127)** | **Uganda (N=8179)** |
| --- | --- | --- |
|  | **N (%)/Median (IQR)** | |
| Post-discharge mortality | 58 (5.2) | 490 (6.0) |
| Age, months | 13.5 (6.1-24.7) | 9 (2.4-19.9) |
| MUAC, mm | 140 (130-158) | 130 (115-145) |
| Weight for age z-score |  |  |
| <-3 | 103 (9.1) | 1131 (13.8) |
| -3 to -2 | 116 (10.3) | 1079 (13.2) |
| >-2 | 908 (80.6) | 5955 (72.8) |
| SpO2, % | 95 (88-97) | 96 (92-99) |
| Time to reach hospital |  |  |
| <30 min | 453 (40.2) | 1821 (22.3) |
| 30 min – 1 hour | 438 (38.9) | 2743 (33.5) |
| ≥1 hour | 236 (20.9) | 3614 (44.2) |
| HIV status | 3 (0.3) | 263 (3.2) |
| How long since last admission |  |  |
| Never | 683 (60.6) | 5495 (67.2) |
| ≤1 month | 233 (20.7) | 893 (10.9) |
| >1 month | 211 (18.7) | 1751 (21.4) |
| Abnormal BCS | 194 (17.2) | 693 (8.5) |
| Respiratory rate | 41 (35-49) | 50 (39-63) |
| Temperature, °C | 37.2 (36.6-38.2) | 37.4 (36.8-38.3) |
| Boil/disinfect/filter water | 441 (39.1) | 5928 (72.5) |
| Water source |  |  |
| Bore hole | 15 (1.3) | 1697 (20.7) |
| Fast running water | 3 (0.3) | 536 (6.6) |
| Municipal water | 715 (63.4) | 3611 (44.1) |
| Open source | 55 (4.9) | 1099 (13.4) |
| Protected spring | 227 (20.1) | 982 (12.0) |
| Slow running water | 42 (3.7) | 251 (3.1) |
| Hemoglobin, g/dL | 11.4 (10.3-12.3) | 11.7 (10.0-13.3) |
| **0-6 months only** | | |
| Duration of present illness |  |  |
| <48 hours | 134 (48.9) | 957 (28.6%) |
| 48 hours to 7 days | 104 (38.0) | 60 (1.8%) |
| 8 days to 1 month | 29 (10.6) | 1985 (59.3%) |
| >1 month | 7 (2.6) | 343 (10.2%) |
| Fontanelle bulging | 5 (1.8) | 132 (3.9%) |
| Neonatal jaundice | 15 (5.5) | 261 (7.8%) |
| Sucking well while breastfeeding | 117 (42.7) | 1956 (58.4%) |
