# Supplementary material for: Validation of a risk-prediction model for pediatric post-discharge mortality after hospital admission for infection in Rwanda: A prospective cohort study
Source: PLOS Glob Public Health. 2025 Jul 1;5(7):e0004606. doi: 10.1371/journal.pgph.0004606 (PMC12212559; doi:10.1371/journal.pgph.0004606)
Supplement: S3 Table — (DOCX) [file pgph.0004606.s003.docx]

**S3 Table:** Rwandan validation cohort characteristics, participant disposition, and post-discharge outcomes at 6 months, stratified by site.

|  | **Kigali (N=361)** | | **Ruhengeri (N=766)** |
| --- | --- | --- | --- |
| **Variable** | **N (%)/Median (IQR)** | | |
| **Demographics** | | | |
| **Sex, n (%)** | | | |
| Female | 124 (34.4) | 327 (42.7) | |
| Male | 237 (65.7) | 439 (57.3) | |
| **Age, months** | 13.3 (4.4-26.6) | 13.7 (6.9-23.8) | |
| **Admission anthropometry** | | | |
| **MUAC (mm)** | 140 (130-160) | 146 (132-156) | |
| <110/<115 | 21 (5.8) | 88 (11.5) | |
| 110-120/115-125 | 49 (13.6) | 63 (8.2) | |
| >120/ >125 | 291 (80.6) | 615 (80.3) | |
| **Weight for age z-score** |  |  | |
| <-3 | 57 (15.8) | 46 (6.0) | |
| -3 to -2 | 42 (11.6) | 74 (9.7) | |
| >-2 | 262 (72.6) | 646 (84.3) | |
| **Admission clinical assessment** | | | |
| **SpO2, %** | 98 (95-100) | 93 (85-96) | |
| **Heart rate** | 142 (128-156) | 142 (128-158) | |
| **Respiratory rate** | 43 (37-51) | 41 (35-48) | |
| **Temperature (°C)** |  |  | |
| < 36.5 | 41 (11.4) | 167 (21.8) | |
| 36.5-37.5 | 121 (33.5) | 298 (38.9) | |
| >37.5 | 199 (55.1) | 301 (39.3) | |
| **Abnormal BCS** | 119 (33.0) | 75 (9.8) | |
| **HIV positive** | 3 (0.8) | 0 | |
| **Positive malaria test** | 10 (2.8) | 7 (0.9) | |
| **Hemoglobin, g/dl** |  |  | |
| No anemia: ≥11g/dL | 188 (52.1) | 524 (68.4) | |
| Anemia: <11g/dL | 173 (47.9) | 242 (31.6) | |
| **Referral** | 269 (74.5) | 741 (96.7) | |
| **Prior antibiotic use** | 265 (73.4) | 158 (20.6) | |
| **Prior antimalarial use** | 31 (8.6) | 2 (0.3) | |
| **Respiratory distress** | 129 (35.7) | 99 (12.9) | |
| **Maternal and Social Characteristics** | | | |
| **Time to reach hospital** |  |  | |
| < 30 min | 92 (25.5) | 361 (47.1) | |
| 30 min – 1 hour | 112 (31.0) | 326 (42.6) | |
| >1 hour | 157 (43.5) | 79 (10.3) | |
| **Water source** |  |  | |
| Municipal water/tap | 182 (50.4) | 533 (69.6) | |
| Other sources | 179 (49.6) | 233 (30.4) | |
| **Boil/disinfect/filter water** | 130 (36.0) | 311 (40.6) | |
| **Maternal education** |  |  | |
| No school or ≤P3 | 38 (10.5) | 113 (14.8) | |
| P4 to P6 | 115 (31.9) | 342 (44.7) | |
| S1 to S6 | 126 (34.9) | 302 (39.4) | |
| > S6 | 79 (21.9) | 9 (1.2) | |
| **Discharge Characteristics** | | | |
| **Discharge status** |  |  | |
| Routine discharge | 343 (95.0) | 754 (98.4) | |
| Referred to higher level of care | 14 (3.9) | 7 (0.9) | |
| Unplanned discharge | 4 (1.1) | 5 (0.7) | |
| **Length of stay** | 6 (3-13) | 4 (2-6) | |
| **Variables collected only for 0-6-month*** | | | |
| **Fontanelle** | 4 (3.6) | 1 (0.6) | |
| **Neonatal jaundice** | 10 (9.1) | 5 (3.1) | |
| **Sucking well when breastfeeding** | 60 (54.6) | 57 (34.8) | |
| **Duration of present illness** |  |  | |
| < 48 hours | 28 (25.5) | 106 (64.6) | |
| 48 hours-7ddays | 52 (47.3) | 52 (31.7) | |
| >7 days | 24 (21.8) | 5 (3.1) | |

Note: * denominator is based on total number in 0-6 months (N =274) and separated by site

|  | **Kigali (N=361)** | **Ruhengeri (N=766)** |
| --- | --- | --- |
| **Outcome** | **N (%)/Median (IQR)** | |
| **Died post-discharge** | 37 (10.3) | 21 (2.7) |
| **Readmission** | | |
| Never | 281 (77.8) | 633 (82.6) |
| Once | 55 (15.2) | 93 (12.1) |
| Twice | 17 (4.7) | 26 (3.4) |
| More than twice | 8 (2.2) | 14 (1.8) |
| **Number of days from discharge to 1^st^ readmission** | 47 (21-86.5) | 57 (25-119) |
| **Number of days from discharge to death** | 42 (20-105) | 16 (4-33) |
| **Location of death*** |  |  |
| At home | 12 (32.4) | 4 (19.1) |
| In-transit | 2 (5.4) | 3 (14.3) |
| In hospital | 23 (62.2) | 14 (66.7) |

Note: * denominator based on total number died after discharge (N=58)

Abbreviations: MUAC: mid-upper arm circumference; SpO2: oxygen saturation; BCS: Blantyre Coma Scale; P: primary; S: secondary
