# Supplementary figures and images for: Validation of a risk-prediction model for pediatric post-discharge mortality after hospital admission for infection in Rwanda: A prospective cohort study
Source: PLOS Glob Public Health. 2025 Jul 1;5(7):e0004606. doi: 10.1371/journal.pgph.0004606 (PMC12212559; doi:10.1371/journal.pgph.0004606)

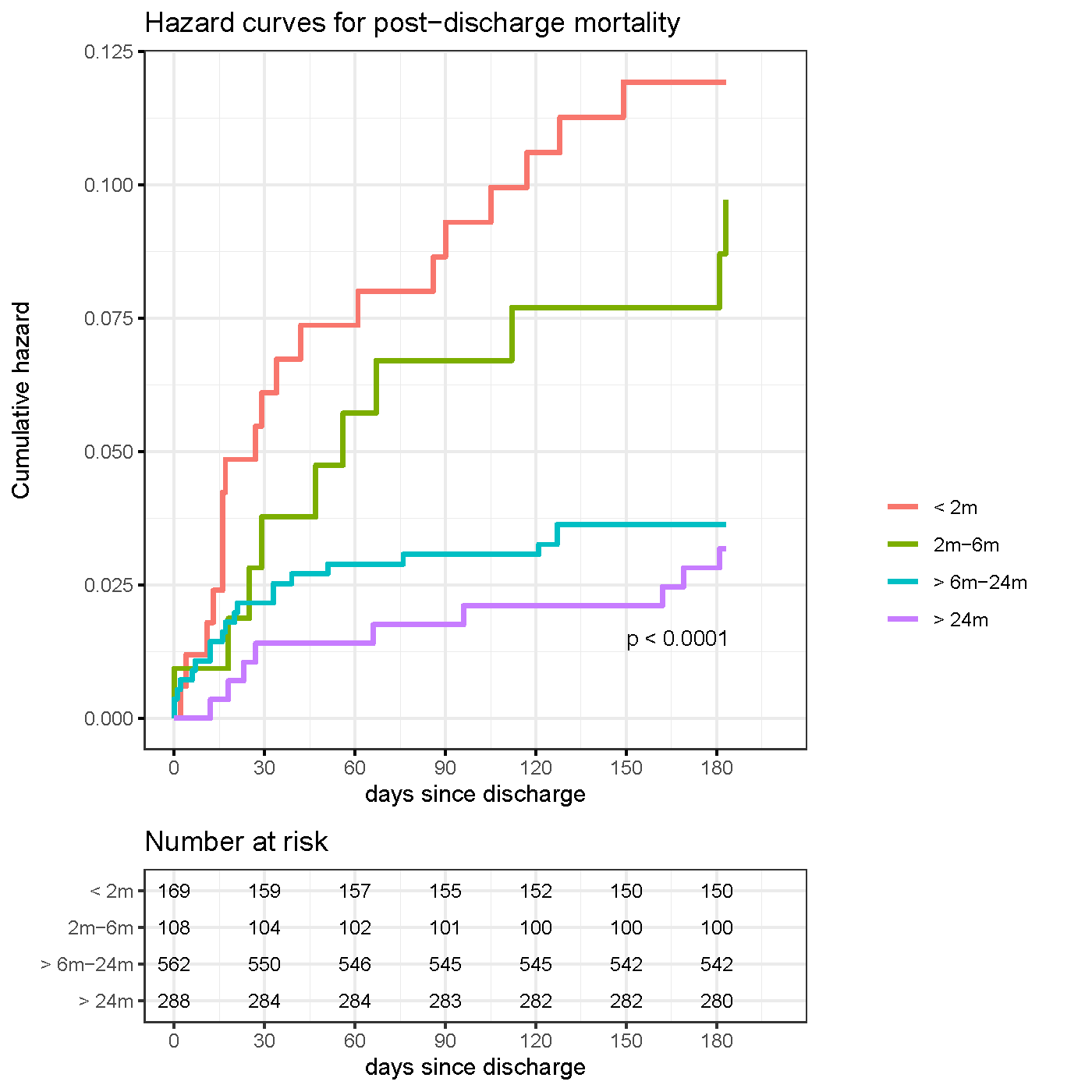

Supplement: S1 Fig — (TIFF) [file pgph.0004606.s005.tiff]

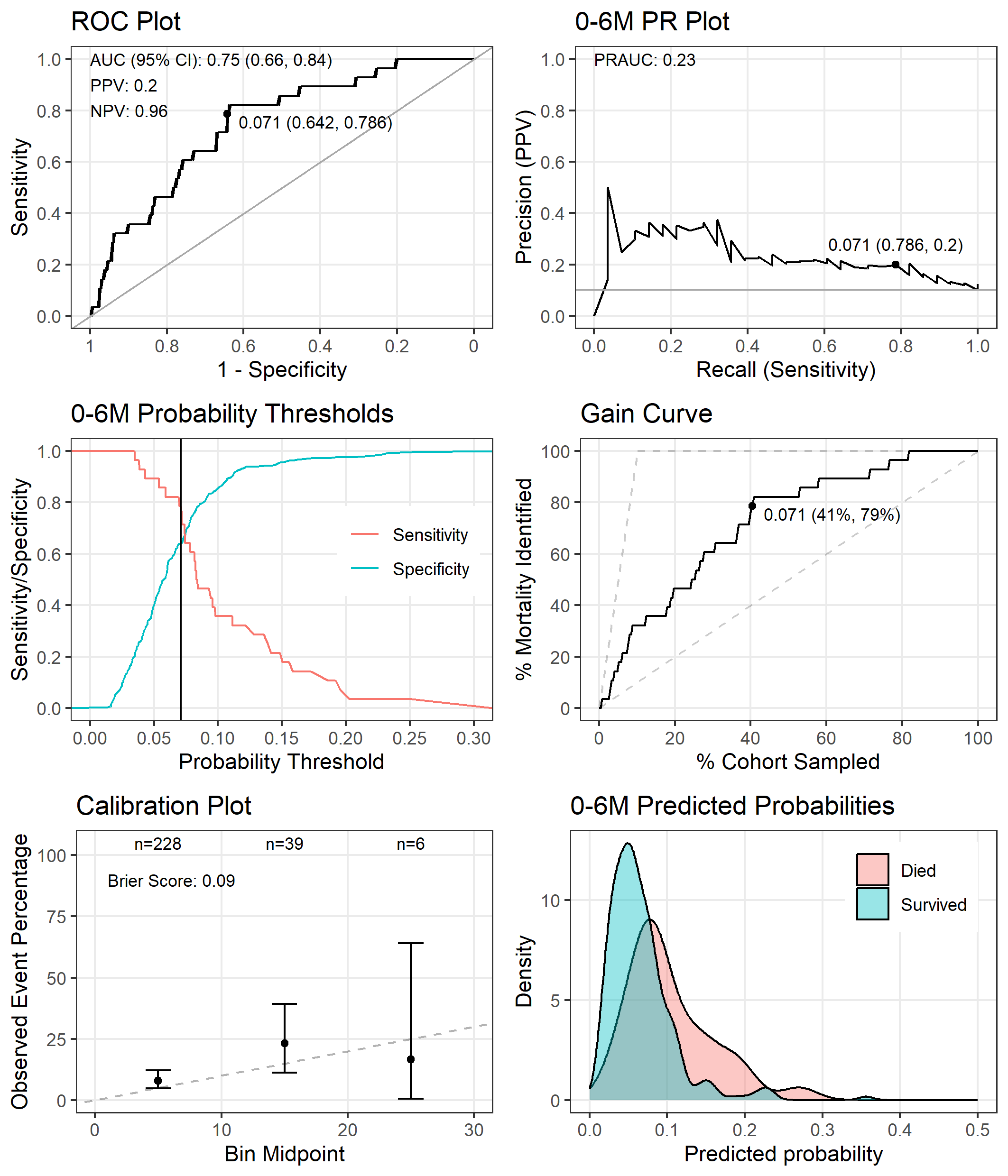

Supplement: S2 Fig — The ROC Plot (a) with the probability of mortality given for the peak AUR; the precision-recall (PR) Plot (b) of model sensitivity by positive predictive value (PPV) to illustrate identification of relevant cases; Probability Thresholds (c) plots outcome probability by sensitivity and specificity, respectively. The Gain Curve (d) depicts % mortality by % of the cohort sampled, and the point shown on the curve is the percentage of participants (starting from the highest risk) needed to be sampled (first percentage) in order to capture 80% of participants with the outcome of interest (second percentage).The calibration plot (e) provides the predicted probability (%) by Observed Event Percentage, with a Brier Score (provided in upper left) closer to 0 indicating greater model accuracy; Predicted Probabilities (f) demonstrates the distribution of predicted probabilities for death and survival given by the model. (TIFF) [file pgph.0004606.s006.tiff]

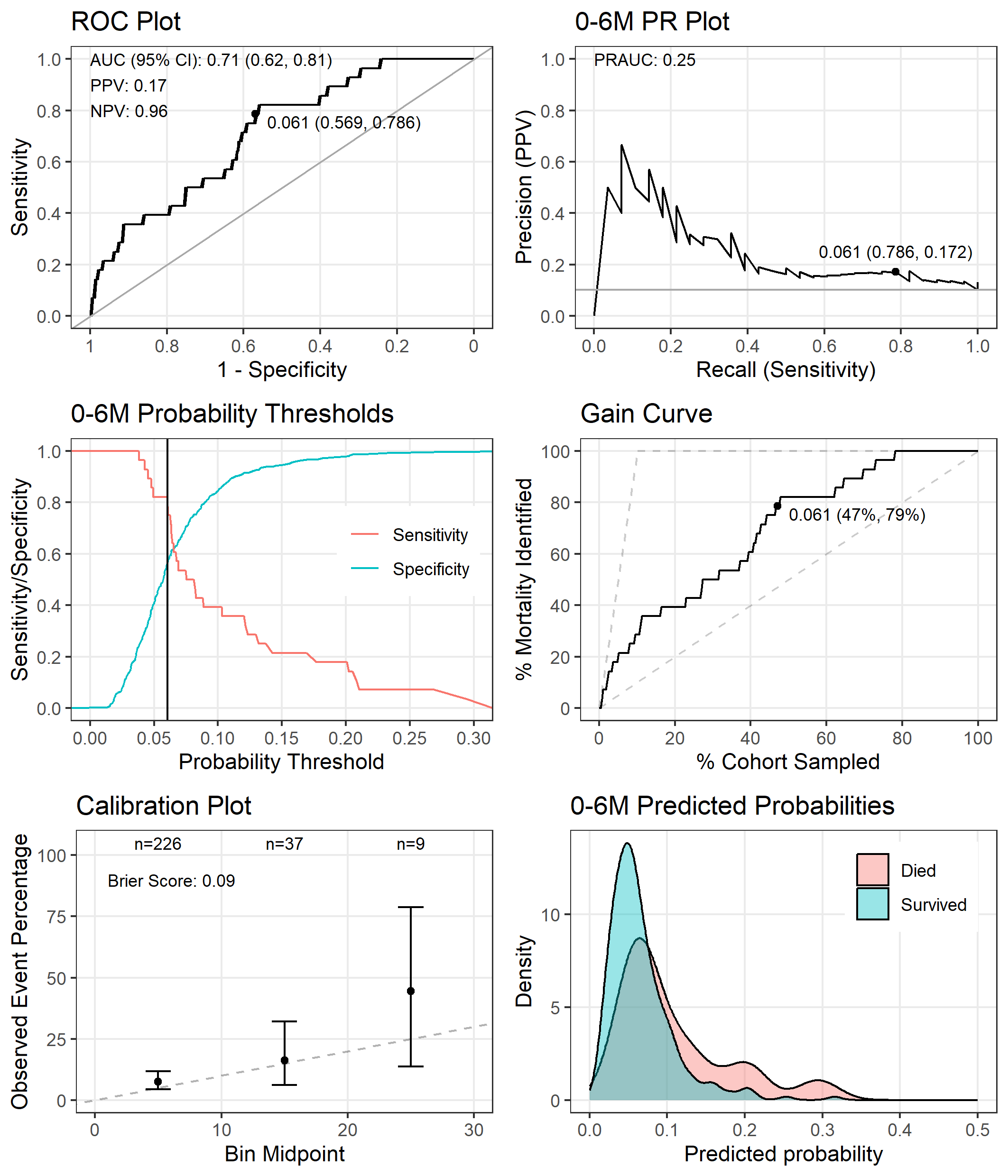

Supplement: S3 Fig — The ROC Plot (a) with the probability of mortality given for the peak AUR; the precision-recall (PR) Plot (b) of model sensitivity by positive predictive value (PPV) to illustrate identification of relevant cases; Probability Thresholds (c) plots outcome probability by sensitivity and specificity, respectively. The Gain Curve (d) depicts % mortality by % of the cohort sampled, and the point shown on the curve is the percentage of participants (starting from the highest risk) needed to be sampled (first percentage) in order to capture 80% of participants with the outcome of interest (second percentage).The calibration plot (e) provides the predicted probability (%) by Observed Event Percentage, with a Brier Score (provided in upper left) closer to 0 indicating greater model accuracy; Predicted Probabilities (f) demonstrates the distribution of predicted probabilities for death and survival given by the model. (TIFF) [file pgph.0004606.s007.tiff]

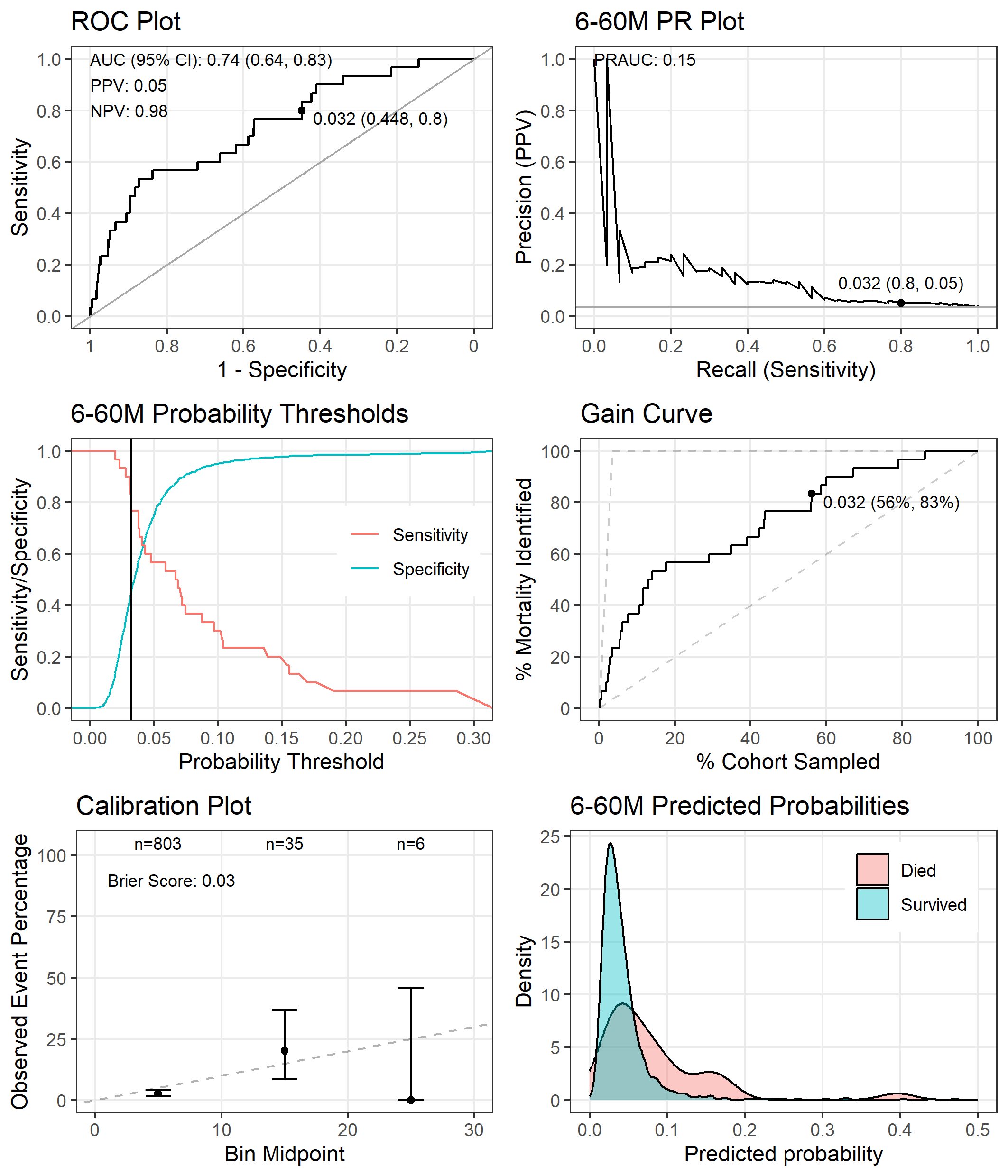

Supplement: S4 Fig — The ROC Plot (a) with the probability of mortality given for the peak AUR; the precision-recall (PR) Plot (b) of model sensitivity by positive predictive value (PPV) to illustrate identification of relevant cases; Probability Thresholds (c) plots outcome probability by sensitivity and specificity, respectively. The Gain Curve (d) depicts % mortality by % of the cohort sampled, and the point shown on the curve is the percentage of participants (starting from the highest risk) needed to be sampled (first percentage) in order to capture 80% of participants with the outcome of interest (second percentage).The calibration plot (e) provides the predicted probability (%) by Observed Event Percentage, with a Brier Score (provided in upper left) closer to 0 indicating greater model accuracy; Predicted Probabilities (f) demonstrates the distribution of predicted probabilities for death and survival given by the model. (TIFF) [file pgph.0004606.s008.tiff]

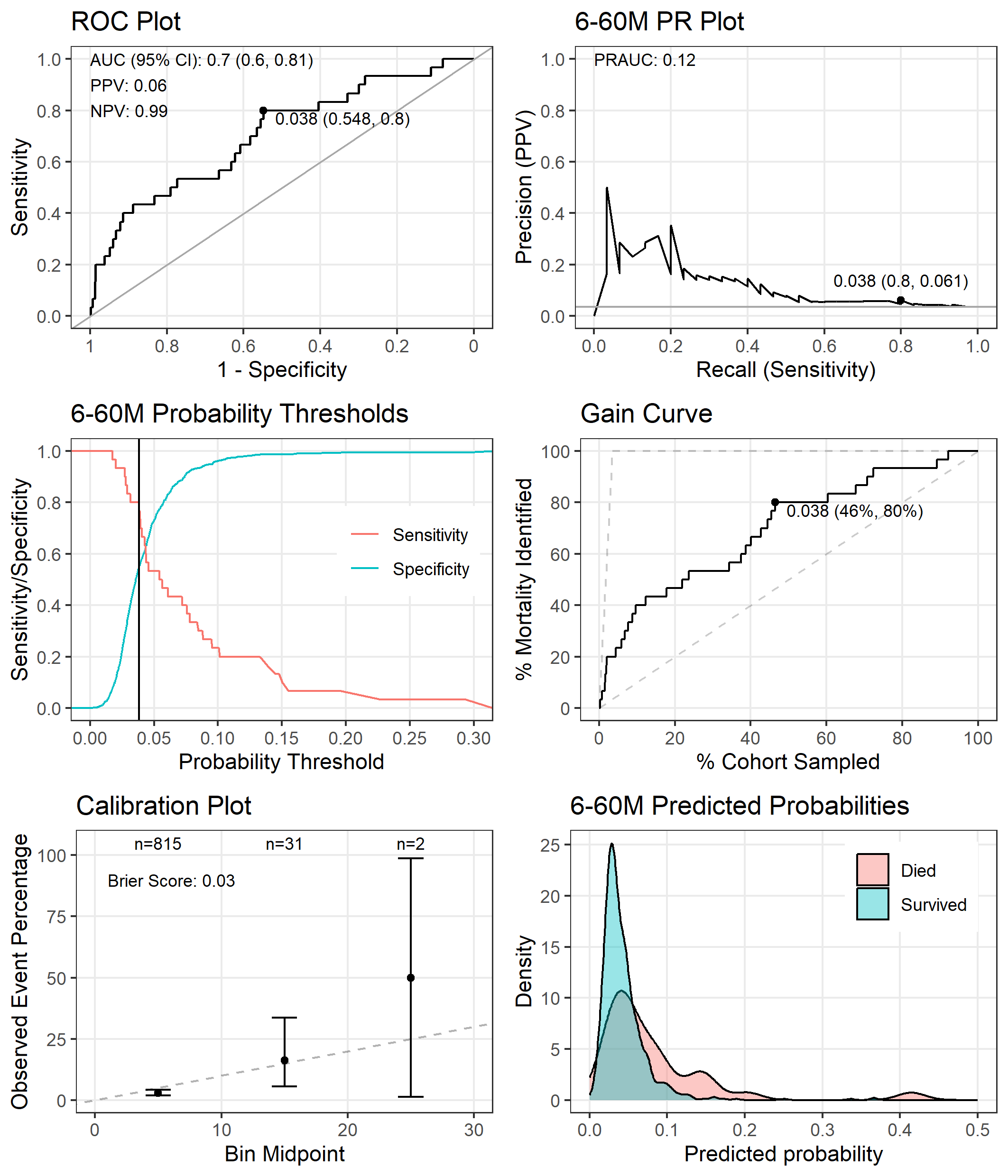

Supplement: S5 Fig — The ROC Plot (a) with the probability of mortality given for the peak AUR; the precision-recall (PR) Plot (b) of model sensitivity by positive predictive value (PPV) to illustrate identification of relevant cases; Probability Thresholds (c) plots outcome probability by sensitivity and specificity, respectively. The Gain Curve (d) depicts % mortality by % of the cohort sampled, and the point shown on the curve is the percentage of participants (starting from the highest risk) needed to be sampled (first percentage) in order to capture 80% of participants with the outcome of interest (second percentage).The calibration plot (e) provides the predicted probability (%) by Observed Event Percentage, with a Brier Score (provided in upper left) closer to 0 indicating greater model accuracy; Predicted Probabilities (f) demonstrates the distribution of predicted probabilities for death and survival given by the model. (TIFF) [file pgph.0004606.s009.tiff]

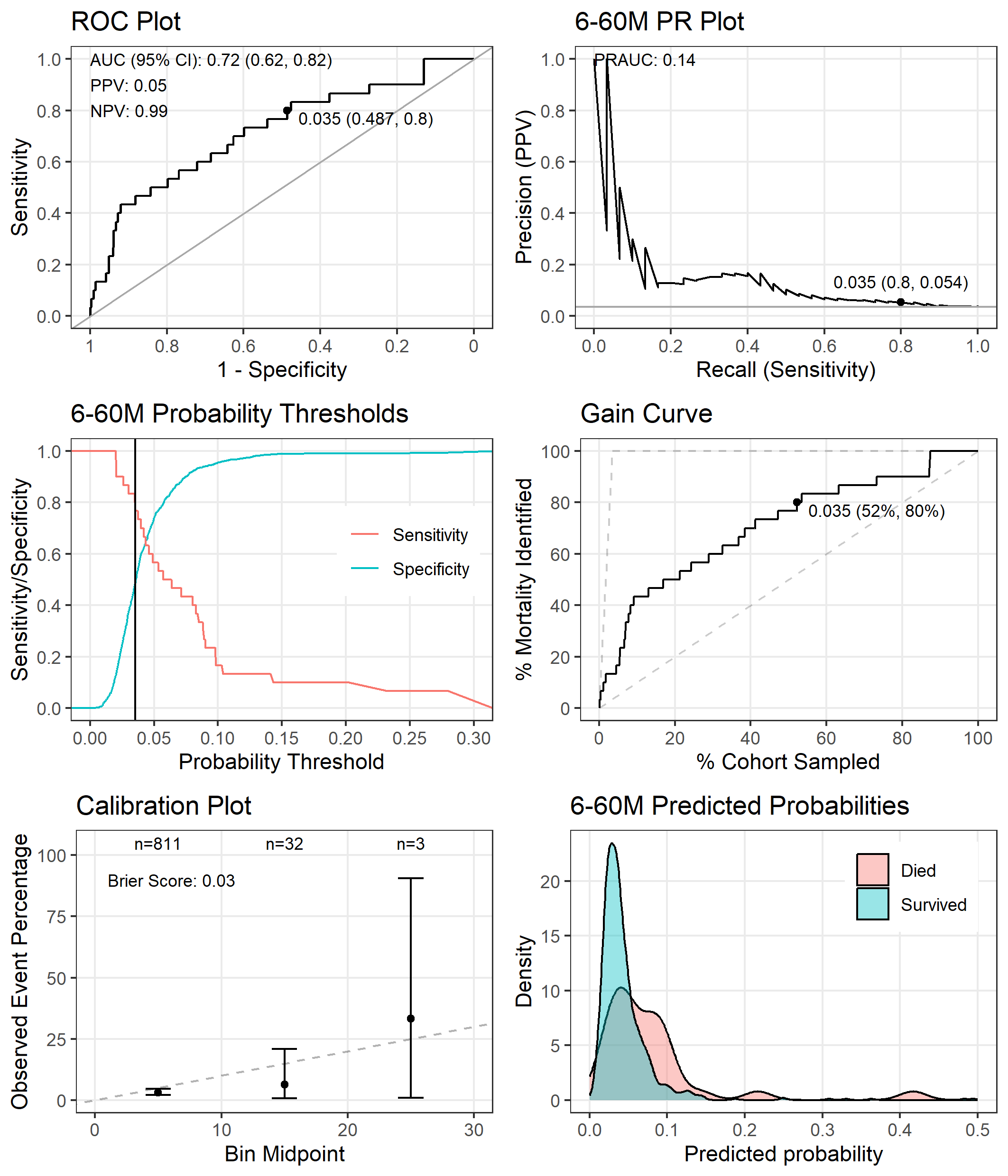

Supplement: S6 Fig — The ROC Plot (a) with the probability of mortality given for the peak AUR; the precision-recall (PR) Plot (b) of model sensitivity by positive predictive value (PPV) to illustrate identification of relevant cases; Probability Thresholds (c) plots outcome probability by sensitivity and specificity, respectively. The Gain Curve (d) depicts % mortality by % of the cohort sampled, and the point shown on the curve is the percentage of participants (starting from the highest risk) needed to be sampled (first percentage) in order to capture 80% of participants with the outcome of interest (second percentage).The calibration plot (e) provides the predicted probability (%) by Observed Event Percentage, with a Brier Score (provided in upper left) closer to 0 indicating greater model accuracy; Predicted Probabilities (f) demonstrates the distribution of predicted probabilities for death and survival given by the model. (TIFF) [file pgph.0004606.s010.tiff]

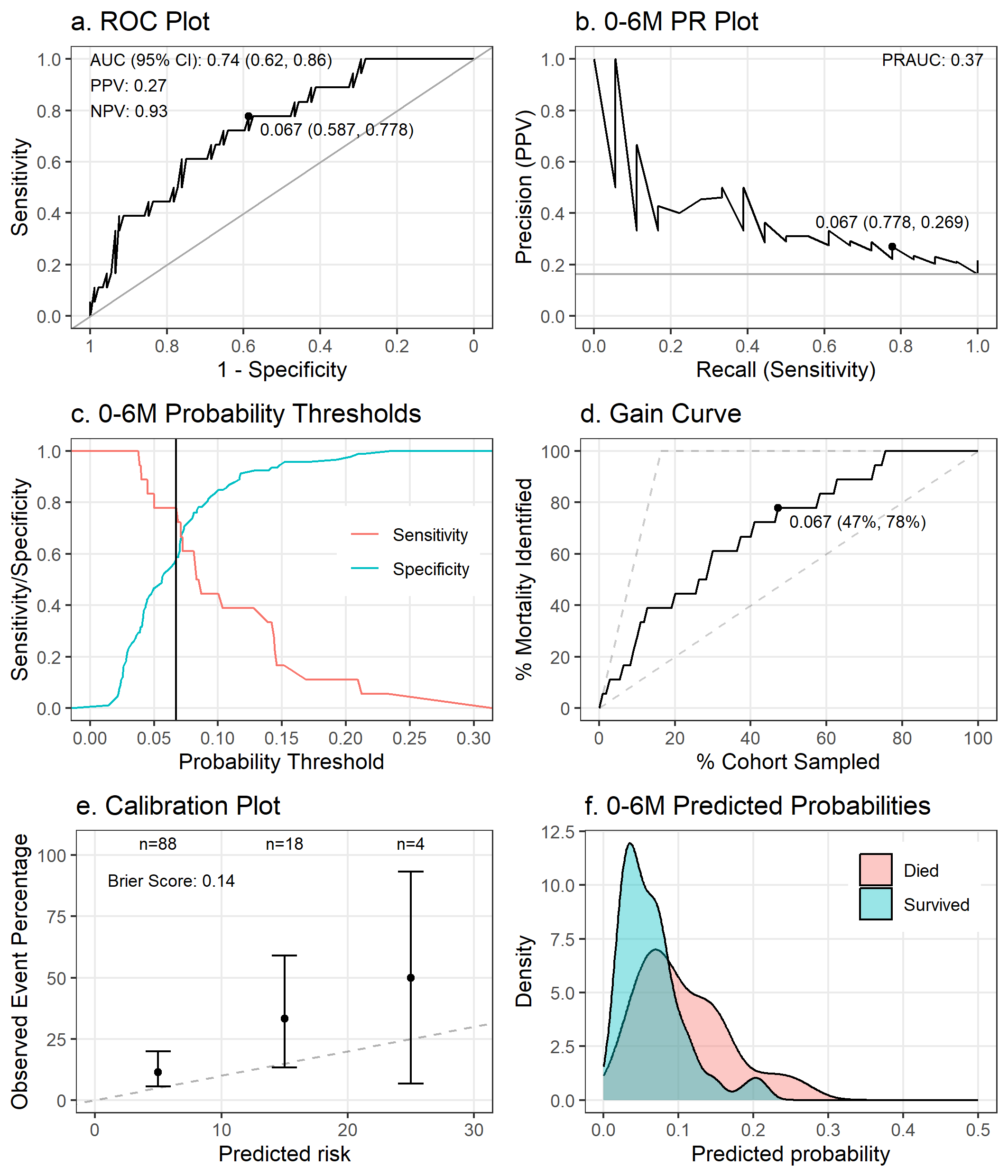

Supplement: S7 Fig — The ROC Plot (a) with the probability of mortality given for the peak AUR; the precision-recall (PR) Plot (b) of model sensitivity by positive predictive value (PPV) to illustrate identification of relevant cases; Probability Thresholds (c) plots outcome probability by sensitivity and specificity, respectively. The Gain Curve (d) depicts % mortality by % of the cohort sampled, and the point shown on the curve is the percentage of participants (starting from the highest risk) needed to be sampled (first percentage) in order to capture 80% of participants with the outcome of interest (second percentage).The calibration plot (e) provides the predicted probability (%) by Observed Event Percentage, with a Brier Score (provided in upper left) closer to 0 indicating greater model accuracy; Predicted Probabilities (f) demonstrates the distribution of predicted probabilities for death and survival given by the model. (TIFF) [file pgph.0004606.s011.tiff]

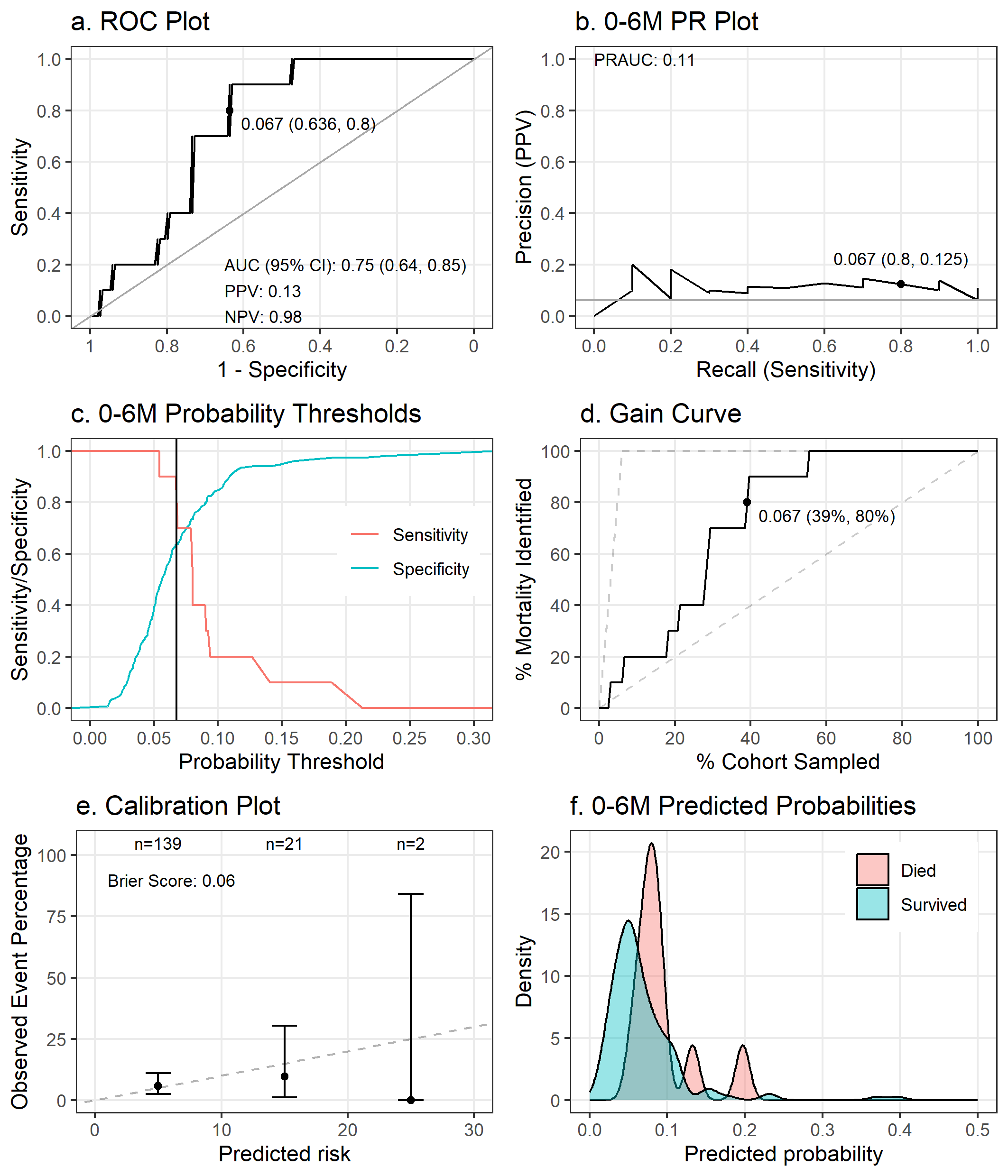

Supplement: S8 Fig — The ROC Plot (a) with the probability of mortality given for the peak AUR; the precision-recall (PR) Plot (b) of model sensitivity by positive predictive value (PPV) to illustrate identification of relevant cases; Probability Thresholds (c) plots outcome probability by sensitivity and specificity, respectively. The Gain Curve (d) depicts % mortality by % of the cohort sampled, and the point shown on the curve is the percentage of participants (starting from the highest risk) needed to be sampled (first percentage) in order to capture 80% of participants with the outcome of interest (second percentage).The calibration plot (e) provides the predicted probability (%) by Observed Event Percentage, with a Brier Score (provided in upper left) closer to 0 indicating greater model accuracy; Predicted Probabilities (f) demonstrates the distribution of predicted probabilities for death and survival given by the model. (TIFF) [file pgph.0004606.s012.tiff]

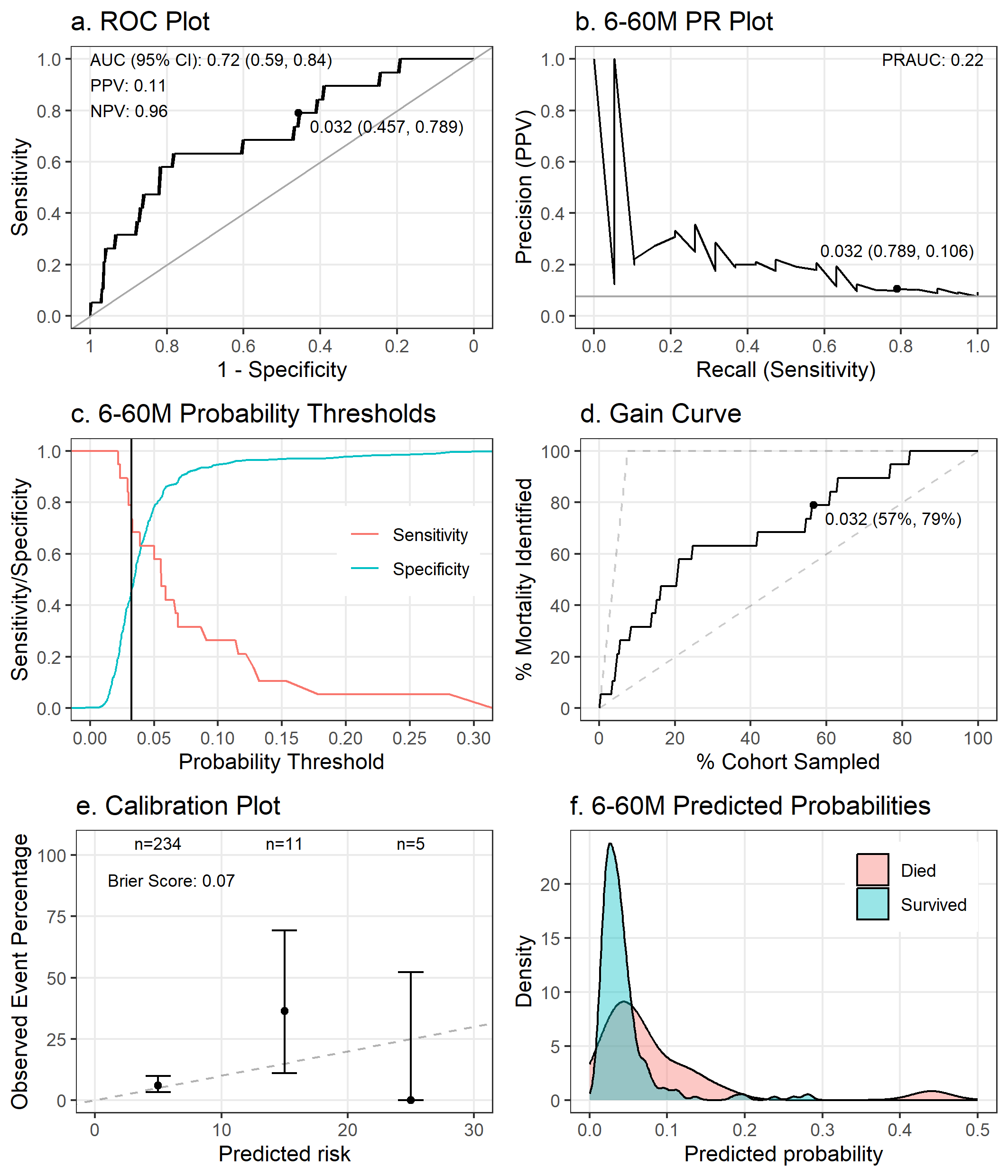

Supplement: S9 Fig — The ROC Plot (a) with the probability of mortality given for the peak AUR; the precision-recall (PR) Plot (b) of model sensitivity by positive predictive value (PPV) to illustrate identification of relevant cases; Probability Thresholds (c) plots outcome probability by sensitivity and specificity, respectively. The Gain Curve (d) depicts % mortality by % of the cohort sampled, and the point shown on the curve is the percentage of participants (starting from the highest risk) needed to be sampled (first percentage) in order to capture 80% of participants with the outcome of interest (second percentage).The calibration plot (e) provides the predicted probability (%) by Observed Event Percentage, with a Brier Score (provided in upper left) closer to 0 indicating greater model accuracy; Predicted Probabilities (f) demonstrates the distribution of predicted probabilities for death and survival given by the model. (TIFF) [file pgph.0004606.s013.tiff]

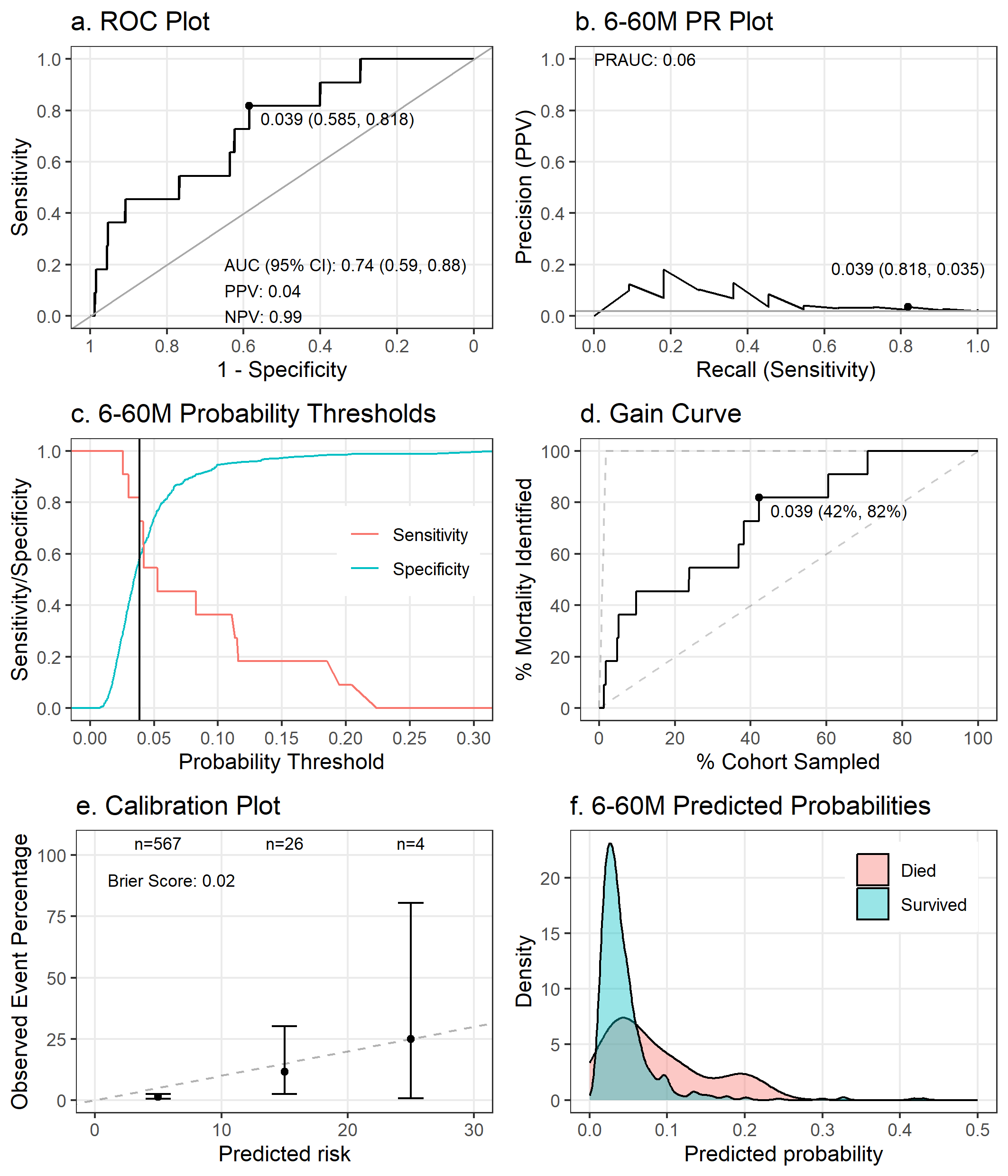

Supplement: S10 Fig — The ROC Plot (a) with the probability of mortality given for the peak AUR; the precision-recall (PR) Plot (b) of model sensitivity by positive predictive value (PPV) to illustrate identification of relevant cases; Probability Thresholds (c) plots outcome probability by sensitivity and specificity, respectively. The Gain Curve (d) depicts % mortality by % of the cohort sampled, and the point shown on the curve is the percentage of participants (starting from the highest risk) needed to be sampled (first percentage) in order to capture 80% of participants with the outcome of interest (second percentage).The calibration plot (e) provides the predicted probability (%) by Observed Event Percentage, with a Brier Score (provided in upper left) closer to 0 indicating greater model accuracy; Predicted Probabilities (f) demonstrates the distribution of predicted probabilities for death and survival given by the model. Abbreviations: ROC: receiver-operator characteristic, AUC: area under the curve, PPV: positive predictive value, NPV: negative predictive value, PR: Precision-recall. (TIFF) [file pgph.0004606.s014.tiff]
