# Supplementary material for: Validation of a risk-prediction model for pediatric post-discharge mortality after hospital admission for infection in Rwanda: A prospective cohort study
Source: PLOS Glob Public Health. 2025 Jul 1;5(7):e0004606. doi: 10.1371/journal.pgph.0004606 (PMC12212559; doi:10.1371/journal.pgph.0004606)
Supplement: Attachment — Submitted filename: Response to reviewers.docx [file pgph.0004606.s015.docx]

April 16, 2025

Dear Editors of PLoS Global Health,

Thank you for the helpful comments and the time spent reviewing our submission. Below, please find an itemized list of comments, organized by reviewer. Below each comment, we provide a response and noted where changes, if relevant, have been made in the manuscript. In some cases, line numbers have changed as a result of modifications to the document, and we note the revised line numbers in the Track Changes version which reflect the edits suggested in each comment.

We have also addressed the following editor’s comments and journal formatting requests:

1. PLOS’ questionnaire on inclusivity in global research: This has been included in the resubmission.
2. Request for exemption of free sharing of code has been requested from the editors as this is housed with the data in the moderated access repository.
3. Figures have been uploaded in the appropriate format.
4. Tables have been added to the document.
5. Permission for exemption of full release of data in a public repository has been emailed to the editors.

We thank you again for your time dedicated to reviewing our work. Please do not hesitate to reach out if there are further questions or clarifications needed.

Sincerely,

Anneka Hooft

[Anneka.hooft@ucsf.edu](mailto:Anneka.hooft@ucsf.edu)

Responses to reviewer comments:

**Reviewer 1:**

Major comments

Abstract:
**line 27. It would more informative for the reader if the Smart discharge risk prediction model is explained more generally. Currently it assumes the reader knows what the author's mean by Smart discharge.**

We apologize for not adding more detail. We have added this to the abstract with some details on variables included in Line 31. Additional detail has been added in the introduction (see below) to provide more information prior models and more distinct reference to the existing publications that detail this work further for those who wish to pursue further explanation.

This portion of the abstract now reads:

*“In Uganda, the Smart Discharges risk-prediction models use simple clinical, socio-behavioral, and point-of-care lab test variables to successfully predict children at the highest risk of death after hospital admission for infection to guide a risk-based approach to post-discharge care.”*

**line 29. The methods in this section need to be explained more. Currently all we know from the abstract is that the models were validated, but the statistical methods are unknown.**

We thank you for noting the need for further detail in the abstract. This has been revised to add additional detail on the validation methods in lines 35-38.

This now reads:

*“We externally validated five models derived from Smart Discharges Uganda in a new cohort of children ages 0 days to 60 months admitted for suspected sepsis at two hospitals in Rwanda. We evaluated model performance using metrics including area under the receiver operating characteristic curve (AUROC), Brier score, and test characteristics (e.g. sensitivity, specificity). Performance was visualized through ROC and gain curves and calibration plots.”*

**line 54. It would be useful to briefly describe in more depth the "socio-behavioural factors linked to increased risk".**

We are happy to provide this. We have added examples of these variables on line 60. Further detail on which factors are included in the actual models can also be found in the list of model components included in Table 2, which is now included and referenced in the manuscript.

This now says:

*“and socio-behavioral factors (e.g. maternal education level, travel time to hospital) linked to increased risk.”*

**line 65. Please elaborate what we mean by variables present on admission? are these signs and symptoms or socio-demographic variables?**
We apologize for the confusion. These include both clinical and socio-demographic variables that are taken at the time the child is admitted. We have added this detail in lines 72-75.

This section now includes the following:

*“The Smart Discharges models use demographic, socio-behavioral, and clinical variables present at the time of admission to predict mortality from infectious causes within the first 6 months following discharge.”*

Methods

**Line 90. The criteria for admission needs greater clarity. Please elaborate on "suspected or proven infection".**
We appreciate you pointing out this need for additional detail. These criteria are purposefully broad given the limited availability of diagnostics and confirmatory testing in this setting. This has been elaborated upon in Lines 112-114 to provide some examples of these infections and to explain that the criteria are based on the judgement of the evaluating clinician.

This text now reads

*“Children aged 0 days to 5 years admitted for suspected (e.g. clinical diagnosis of pneumonia) or proven (e.g. malaria test positive) infection as determined by the admitting clinician at the time of admission (13) were considered for enrollment.”*

Validation of Smart Discharges Models

**line 144. There no mention of the types of prediction models used in this study. A brief description is essential to understand the performance of the model.**

**similarly, it is not clear which variables were used in the models from the text. One has to review the supplementary material to ascertain this.**

Please forgive our oversight as the lack of inclusion of the larger table files helps to clarify this. We have now added in the tables which provide the individual model components. Detail on the models is included in lines 173-178, which we hope is now easier to follow after the inclusion of the proper table for reference. Line 169 has been updated to reflect this information can be found in Table 2, not Table 1. Additional detail on model development and explanation of which components were included has also been added to the methods in Lines 80-93 to add the following for clarification:

*“Briefly, the original models were derived as follows: potential components were chosen a priori through a modified Delphi process and grouped into categories. We then used elastic net regression to create 3 different model types from the pool of available variables; one model using common clinical variables (e.g. pulse oximetry); one model using on both clinical and social variables (e.g. water source); and one model using any predictor variable (e.g. point-of-care lab test results) to allow for flexible use during implementation pending available resources and data. We then used the weighted sums of the absolute regression of each variable to rank importance and selected the top 8 eight unique variables for inclusion in the final model. Models were limited to 8 components given the anticipated challenge of using larger models during implementation in real-world settings. Separate sets of models were developed for children under 6-months of age and those 6-60 months of age, to accommodate for age-related heterogeneity in outcome prediction. See Wiens 2024 for full details on this process.(12) Model components can be found in Table 2.”*

**line 159. Please describe the justification for the 80% cut-off in more depth.**
We are happy to provide further explanation. This was decided upon through meetings with key health leaders and policymakers upon discussion of the results of the original studies in Uganda. This cutoff was determined to adequately balance the need for robust detection of this serious outcome while avoiding overburdening already constrained systems with over-detection of lower risk cases. This has been explained further in lines 187-188 as it now reads:

*“For this analysis, a sensitivity threshold was set at 80% based on our prior work and discussions with key stakeholders and determined as the ideal cut point to balance resource prioritization while minimizing risk of a severe, high-risk outcome.”*

**line 162. Please describe more fully how missing data was investigated. What was the distribution of missing variables more generally and were these assumed to be missing at random?**

**Similarly, was this done before or after the data were split.**

We investigated missing data by tabulating each variable and put the distribution in Table 1. This was done for the whole and split data. They were assumed to be random and we used Little’s test to check for that assumption. We have incorporated this into lines 191-194 of the manuscript. This now reads:

*“We investigated missing values through tabulation of each variable (distribution shown in Table 1) for both the whole dataset and by age group. Missing data was assumed to be random, with this assumption confirmed using Little’s test and k-nearest neighbor was used for the imputation of missing values.”*

Results

**Table 1 and Table 2 appear to be missing from the document.**

Apologies, these were included as separate files due to their large size and were not added. They have now been added to the manuscript text.

**line 179. Please consider including confidence intervals for the model degradation percentages to better contextualize the findings**

We have considered this request but given the nature of this calculation, this is not possible. We have added the model degradation percentages to Table 2 and indicated in Lines 217-218 of the results that none of these percentages had a significant p-value.

To get the percentage of model degradation, we use the difference in area under the curve (AUC) between the validation set and the original model divided by the new model: AUC1-AUC2)/AUC2. It is possible to calculate a confidence interval (CI) for the absolute difference between AUC1 and AUC2, but not for the percentage as we would need two sets of Cis, and it is not possible to have two sets of CIs for one difference that is calculated. The common practice is to obtain a p-value to test for significance of degradation, which we have done; none of which were found to be significant.

**The sample size was estimated at 8% post-discharge mortality rate, but the observed rate was lower (4.8%), which may have impacted the study’s power. Please elaborate on this in the discussion.**

The low outcome frequency is discussed in study limitations, including its limitations on power to calculate risk levels beyond 10%. We understand that a lower observed mortality rate and reduced power decreases reliability around our point estimates and widens confidence intervals of our validity measures. This may make it more difficult to rule out potentially important differences. We have added an additional phrase to lines 311-313 to make this clear so it now reads:

*“First, this study observed few outcomes, limiting the inferential strengths of the validation results, thus, this may make it more difficult to rule out potentially important differences.”*

Discussion:

**The discussion would benefit from more detailed exploration of how these models could be integrated into routine clinical care, particularly given the authors' appropriate concerns about data quality when collected outside research settings.**

We thank you for highlighting this. This has been introduced in Lines 308-310. We have added lines 322-323 to further emphasize the need for implementation studies to assess integration of these tools into routine clinical practice in real world settings.

These additions read as follows:

*“Furthermore, implementation outcomes such as feasibility, accessibility, fidelity, and impacts on cost and other markers of care quality and resource utilization will need to be further elucidated to understand use and optimize uptake in real world settings.”*

And

*“Finally, these models were validated using prospective research data, which may not reflect the data captured during routine care by facility healthcare staff. In such settings, these models may reflect an optimistic scenario, both regarding data quality and completeness, hence the need for future studies assessing implementation outcomes in real-world settings.”*
**Some consideration of the cost implications of implementing risk-stratified care would be valuable.**

We agree that this is an extremely important consideration of any study in this context. This concept has also been introduced, but because this study only explored the prediction model, further interventional studies are needed to determine cost implications as addressing mortality risk will be where the majority of costs are incurred. We have added discussion on the need for cost assessment studies in lines 325-326 so that it now includes:

*“In addition, cost of routine integration of risk-stratified care into clinical practice and the implications of risk reduction interventions also needs to be explored.”*

We also added additional information on the potential impacts of increasing follow-up on a those identified to be high-risk in lines 281-284 which also considers potential cost and reads as follows:

*“Given the serious nature of this health outcome, even in a resource-limited setting, follow-up of 50% of high-risk children is still likely to be highly feasible and cost-effective, given the possible prevention of more costly readmissions and utilization of simple interventions such as improved caregiver education at discharge or follow-up visits at community-based health centers.”*

Figures and Tables
**Fig 1 and 2, could benefit from clearer labelling and captions to enhance accessibility for non-specialist readers. Ideally these should stand alone.**

We apologize for this lack of detail. Additional detail has been added to both figure legends.

Reviewer #2:

**1.In the title: I recommend to mention that the model only concerns post-discharge mortality for suspected sepsis (not all cause hospitalisations).**

We thank you for noting this and did not mean to mislead. We have added “infection” to the title to specify this as requested.

The title now reads as:

*“Validation of a risk-prediction model for pediatric post-discharge mortality after hospital admission for infection in Rwanda”*
**2. Line 90: ‘Suspected or proven infection’: it would be useful to specify which criteria define a ‘suspected or proven infection’, who assesses them (clinician or researcher), and when (on admission, during hospitalisation). Furthermore, line 63 mentions that the model was developed on children admitted with suspected sepsis, and not all children with ‘suspected or proven infection’. I would clarify/rationale the inclusion criteria.**

We apologize for not clarifying this further. We have now added more detail that suspected or proven infection is more general as determined by the admitting clinician. This is to increase inclusivity given the lack of diagnostics available. This has now been changed in Lines 112-114.

We have changed sepsis to infection for consistency in the manuscript and abstract. These terms were used interchangeably and detail is added that prior studies by our team have indicated that most children meeting the criteria of suspected or confirmed infection in these settings also have high frequency of meeting clinical criteria for a diagnosis of sepsis. A reference has been provided for this and these details have been been added in Lines 114-116.

This section now reads as follows:

*“Children aged 0 days to 5 years admitted for suspected (e.g. clinical diagnosis of pneumonia) or proven (e.g. malaria test positive) infection as determined by the admitting clinician at the time of admission (13) were considered for enrollment. Our prior study demonstrated that the majority of these children are considered to meet clinical criteria for sepsis.”*

**3. Clarify whether only day 0 data were included in the score (since vitals/other parameters at discharge were captured)**

Only Day 0 values are included in the model. This has been clarified in line 170.

**4. Line 144: Is there a reference detailing the initial model development? In particular, it would be useful to know how the various predictors are integrated into the initial model (continuous, categorical (if so, number and definitions of categories)).**

We have added in reference on the original model development and a brief explanation of how this was done in Lines 80-92, with the reference to the manuscript with more detail on this also provided.

This passage now includes:

*“Briefly, the original models were derived as follows: potential components were chosen a priori through a modified Delphi process and grouped into categories. We then used elastic net regression to create 3 different model types from the pool of available variables; one model using common clinical variables (e.g. pulse oximetry); one model using on both clinical and social variables (e.g. water source); and one model using any predictor variable (e.g. point-of-care lab test results) to allow for flexible use during implementation pending available resources and data. We then used the weighted sums of the absolute regression of each variable to rank importance and selected the top 8 eight unique variables for inclusion in the final model. Models were limited to 8 components given the anticipated challenge of using larger models during implementation in real-world settings. Separate sets of models were developed for children under 6-months of age and those 6-60 months of age, to accommodate for age-related heterogeneity in outcome prediction. See Wiens 2024 for full details on this process.(12) Model components can be found in Table 2.”*

**5.Line 144 : Reference to Table 1 seems to be erroneous (title does not correspond to line 176)**

This has been changed to Table 2, which provides the model components.

**6.Line 145 : Explain how the risk score is used to calculate the predicted probability of death**

The coefficients from the risk prediction model (provided in Supplementary Table 1) are applied to the child’s individual results to calculate an overall percentage risk of the binary outcome of post-discharge death. This explanation has been added to Lines 172-174. This now includes these additional details:

*“Coefficients for individual model components were applied to each participant’s unique responses for these variables and used to calculate the predicted probability of post-discharge death (e.g. percent likelihood of death) for each participant.”*

**7.Results : The proportion of missing data for predictors and outcome are not mentioned. Since the k-nearest neighbor method was used for the imputation of missing values, were the missing data missing at random?**

We appreciate the attention paid to this detail. We investigated missing data by tabulating each variable. The distribution for this is already included in Table 1. This process was done for the whole and split data. Missing data were assumed to be random and we used Little’s test to check for that assumption. This detail has been added to the Methods in Line 191-194 and reads as follows:

*“We investigated missing values through tabulation of each variable (distribution shown in Table 1) for both the whole dataset and by age group. Missing data was assumed to be random, with this assumption confirmed using Little’s test and k-nearest neighbor was used for the imputation of missing values.”*

**8. Line 184 : Why the specificity is not mentioned ?**

We apologize for this oversight. We had emphasized sensitivity in relation to the outcome, but do think it is also important to provide specificity. We have added this now with the other characteristics in Lines 224-227 and to Table 2.

**9. The study was carried out in 2 different HFs, a university hospital and a district hospital: mortality is probably higher in the university hospital. Would it therefore be possible to specify the results of the primary outcome depending on the hospital? [Comment to be ignored if present in Table 2]. It may also be interesting to have a sensitivity analysis of the model's performance on these sub-populations (Kigali University Hospital versus regional hospital), as the performance may be different.**

We acknowledge this very valid point. We have added this in Supplementary Figures 3-6. Given the small sample size at each hospital we focused on the combined dataset. Stratifying data by hospital still demonstrates reasonably good AUC values. As expected, Brier scores in the smaller groups increased, though still remained adequate. This has been mentioned in the text in lines 242-244, as follows:

*“Model performance was reduced, but remained adequate when stratified by hospital, as expected given the reduced sample size (Supplementary Figures 3-6).”*

**10. Comparison with the development data of the distribution of important variables (demographics, some important factors such as HIV prevalence, predictors and outcome) is missing. It would also have been interesting to mention the type of population on which the Ugandan model was developed (was it a population from district/regional/zonal hospitals), and to compare it with the validation population in Rwanda.**

We appreciate the attention paid to the current study in the context of the original model derivation population. We have now added a table comparing the two study populations as Supplementary Table 2. These included both urban and rural hospitals in Uganda, most of which were regional referral hospitals. Significant variations between the two groups are mentioned in Lines 206-208 of the text. This reads as follows:

*“Overall mortality rate was comparable to that of the Ugandan derivation cohort (5.2% vs. 6% respectively), with comparison between the two groups provided in Supplementary Table 2. Of note, prevalence of HIV and malnutrition were notably lower in the current cohort.”*

**11.Discussion: It seems important to discuss the low positive predictive value at 6-60 months, which implies many ‘false positives’: discuss the potential impact in term of resources to have to provide additional follow-up for 50% of discharged children in a context of limited resources.**

Given the high-risk nature of this outcome, prioritization was made toward maximizing sensitivity, understanding this would lead to potential for increased false positive results. Potential interventions for these children can be as minimal as enhanced discharge education to the caregiver and follow up in their community health center. In conversations with healthcare administration and leadership in these areas, they agreed that that this tradeoff was reasonable. In addition, in our epidemiologic studies of post-discharge death have demonstrated that many of these children seek care or are readmitted prior to death, indicating that improved risk prediction earlier on may actually help with some conservation of resources (e.g. outpatient follow up vs. need for repeat, more costly readmission). This will need to be explored further in interventional studies with cost analysis.

These explanations have been added to the discussion in lines 281-284 and now includes this passage:

*“Given the serious nature of this health outcome, even in a resource-limited setting, follow-up of 50% of high-risk children is still likely to be highly feasible and cost-effective, given the possible prevention of more costly readmissions and utilization of simple interventions such as improved caregiver education at discharge or follow-up visits at community-based health centers.”*

**12. Line 252: discuss the low number of patients with outcomes (n=58) (below the common rule of thumb to have at least 100 patients with the outcome of interest in the validation dataset to ensure reliable estimates of model performance + below the number estimated in the sample size calculation)**

We understand this concern. The discussion includes mention of how the overall low rate of outcomes may affect results. We have added an additional phrase to lines 311-313 to further emphasize this point and the effects it may have on the estimates of model performance so it now reads:

*“First, this study observed few outcomes, limiting the inferential strengths of the validation results, thus, this may make it more difficult to rule out potentially important differences.”*

**13. Limitation: Consider mentioning that it is not a bedside model (not easy-to-use simplified model-requires an electronic device)**

We have added this to the limitations, noting that this may become less of an issue as more clinicians use smartphones or other electronic devices at the bedside, and as even LMICs transition to electronic medical records systems. This has been added in lines 325-330. This includes the following:

*“In addition, cost of routine integration of risk-stratified care into clinical practice and the implications of risk reduction interventions also needs to be explored. We also acknowledge that the tool requires use of an electronic device, however, we feel this is becoming increasingly feasible as this tool can easily be utilized on a phone-based app by a provider at the bedside without need for internet connection or integrated into an electronic medical records system as more countries, including Rwanda, push toward digitalization of care.”*

**14. Fig 2: Rephrase ‘bin midpoint’ on the x-axis**

This has been changed to “% Probability”
